# Schizophrenia-spectrum patients treated with long-acting injectable risperidone in real-life clinical settings: functional recovery in remitted versus stable, non-remitted patients (the EVeREST prospective observational cohort study)

**DOI:** 10.1186/s12888-016-0712-1

**Published:** 2016-01-15

**Authors:** Elisabeth Giraud-Baro, Daniel Dassa, Florent De Vathaire, Ricardo P. Garay, Joelle Obeid

**Affiliations:** Hospital Center of Saint-Egrève, Saint-Egrève, France; CHU La Timone, Marseilles, France; INSERM U1018, Institute Gustave Roussy, Villejuif, France; Craven, Villemoisson-sur-Orge, France; Janssen-Cilag France, Issy Les Moulineaux, France

**Keywords:** Functioning, Schizophrenia, Social functioning, Risperidone, Risperidone long-acting injection

## Abstract

**Background:**

Previous studies showed functional improvement in *stable* patients with schizophrenia treated with risperidone long-acting injection (LAI). We therefore re-investigated functional improvement with risperidone LAI in *remitted* patients, in comparison with *stable* patients. The study was conducted in real-life conditions because of the high heterogeneity of the patients’ situations.

**Method:**

This was a multi-centre, prospective observational cohort study involving adult schizophrenia-spectrum chronic patients who were previously treated with risperidone LAI for 6 months. Remission was evaluated using the consensus criteria proposed by the Remission in Schizophrenia Working Group (RSWG). The primary endpoint was global functioning (assessed with the Global Assessment of Functioning scale, GAF) after one year of treatment. Social functioning was a secondary outcome.

**Results:**

The analysis included 1490 patients. Attrition rate was 9.1 % at the end of the study. 27.7 % of patients were in remission after one year of risperidone LAI treatment. The mean GAF rating score (62.5 ± 1.5) was higher than the cut-off previously used to identify patients with satisfactory functioning (60) and significantly higher than the mean GAF score in stable, non-remitted patients (48.3, *p* < 0.001). Social functioning was also high in remitted patients (21.0 ± 3.6 vs. 17.2 ± 3.7 in non-remitted patients, *p* < 0.001).

**Conclusion:**

The results clearly show that after one year of treatment with risperidone LAI, RSWG-remitted patients have a high level of global functioning, which is significantly higher than in stable, non-remitted patients. Social functioning was also higher in remitted patients as compared with stable, non-remitted patients.

## Background

A long-acting injectable (LAI) form of the atypical antipsychotic risperidone was introduced 12 years ago to reduce the risk of partial compliance and adverse outcomes in patients with schizophrenia [1–3]. The symptomatic efficacy of risperidone LAI was evaluated using the consensus criteria proposed by the Remission in Schizophrenia Working Group (RSWG; a state of no greater than low-to-mild intensity in core psychotic symptoms, sustained for a minimum duration of 6 months) [4]. These studies showed higher remission rates with risperidone LAI (21–45 %) [5–8] as compared with oral antipsychotics (between 6.3 % for risperidone and 12.4 % for olanzapine) [9].

The ultimate goal of treatment in schizophrenia is recovery, i.e., patients regaining functioning and participating in social and vocational opportunities [10]. Previous interventional studies showed functional improvement under risperidone LAI treatment in patients with non-acute, *stable* schizophrenia or schizoaffective disorder [11–14]. On the other hand, a post-hoc analysis of the StoRMi (Switch To Risperidone Microspheres) interventional trial showed both, global functioning recovery and RSWG-remission in 21 % of patients under risperidone LAI [15]. Finally, the therapeutic benefit of drugs should be re-examined in real-life conditions because of the high heterogeneity of the patients’ situations.

Taking all the above elements together, we re-investigated functional improvement with risperidone LAI in *RSWG-remitted* patients, in comparison with *stable* patients. The study was conducted in real-life conditions because of the high heterogeneity of the patients’ situations (the EVeREST, EValuation of functioning in REmission after Symptomatic Treatment, study). Although the Global Assessment of Functioning (GAF) scale is widely used to evaluate functioning, we decided to use an additional scale, to evaluate social skills and social roles (“ad hoc” scale shown in Table 1).

**Table 1:** Social Functioning in Schizophrenia scale

Item	Characteristics
Social skills	
Personal care and appearance	Personal hygiene and clothing
Housekeeping	Housework, laundry, shopping (food, etc.)
Familial and social integration	Relationships necessary to maintain harmonious integration
Information and execution	Ability to get information and fulfill administrative and social formalities of everyday life
Social roles	
Organizing free time	Ability to engage in social, creative or recreational activities (attendance at social groups, foundations, clubs or groups of mutual aid)
Managing stigmatization	Knowing one’s disability, asserting rights, withstanding criticisms
Working^a^	Ability to project into professional life according to the degree of disability. Having a job in a protected or ordinary environment

*Scale.* 1: do not do – 2: neglect – 3: do, but with efforts – 4: do without effort

^*a*^ *Scale.* 1: not applicable – 2: projects of professional life – 3: in training for working rhythms – 4: has a job in a protected or ordinary environment

## Methods

### Study design

**Fig. 1:**
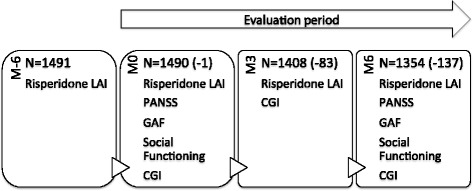
Clinical study flow diagram. Number of patients is given with dropout numbers in brackets. M-6 (risperidone LAI initiation, 6 months before inclusion), M0 (month 0, first evaluation visit), M3 (month 3, follow-up visit), M6 (month 6, end of study)

This was a multi-centre, prospective observational cohort study of 1554 adult schizophrenia patients who were initiated to risperidone LAI treatment 5 to 7 months before the beginning of the study (Fig. 1). Data was communicated by 381 French psychiatrists, between July and December 2008. The study was sponsored by Janssen Cilag (Issy les Moulineaux, France). C2R (Paris, France) was in charge of the setting-up of the study and provided access to the TVF database of CEGEDIM (Centre de gestion et de Documentation de l’Information Médicale; Management and documentation centre of medical information, Boulogne-Billancourt, France). STATITEC (Labege, France) was in charge of the statistical analysis.

The study was declared to the CNIL (Commission Nationale de l’Informatique et des Libertés; National Committee of informatics and Freedom) and authorized by the CCTIRS (Comité Consultatif pour le Traitement de l’Information en matière de Recherche dans le domaine de la Santé; Advisory Committee for the Evaluation of Information in the field of Health Research) [16]. Patients were informed about the study by the participant psychiatrists and gave consent to communicate data. The study also agreed with the Helsinki Declaration (1975, revised 1983) (World Medical Association Declaration of Helsinki: http://www.wma.net/en/30publications/10policies/b3/index.html).

The number of French psychiatrists practicing in a public or private structures for management of psychotic patients, including patients with schizophrenia, was estimated at 5500 [17]. A list of 1500 physicians, balanced and stratified by region, was selected at random by using the TVF database to obtain a representative sample at the national level. This database, which is commonly used by French Health Services, is under the control of the CNIL. The psychiatrists interested in participating were contacted by regular mail to obtain a written agreement to take part in the study and certify their knowledge in safety procedures concerning serious adverse events.

The study included adult patients (18–65 years old) diagnosed as schizophreniform or schizophrenia disorder (DSM-IV criteria) who had been under treatment with risperidone LAI for five to seven months and who had decided to continue treatment (Fig. 1, M-6). To ensure representativeness of the studied population, only the first three consulting patients were included. The evaluation period was six months and included three visits (Fig. 1): visit 1 (M0, month 0; 6 months after risperidone LAI initiation), visit 2 (M3, month 3; 9 months after risperidone LAI initiation), and visit 3 (M6, month 6; at the end of the study, 12 months after the initiation of risperidone LAI treatment).

At visit 1, 70.8 % of patients had an adjunctive drug treatment (anxiolytics 57.6 % of patients, hypnotics 41.8 %, antidepressants 30 %, antiparkinsonians 21.3 % and mood stabilizers 21.0 %). Moreover, 55.7 % of patients received psychosocial interventions.

### Data collection

The participating psychiatrists collected the following patients’ information (in case report forms):

- ➢ At M0
  - ✓ Demographic aspects
  - ✓ History of schizophrenia or schizophreniform disorder
  - ✓ Actual clinical and therapeutic aspects, including:
    - Treatment follow-up (number of missed injections, date and reason for discontinuation of treatment, if necessary)
    - PANSS (positive and negative symptom scale) core of psychotic symptoms
    - Functioning, assessed by the Global Assessment of Functioning (GAF) scale and by a Social Functioning in Schizophrenia “ad hoc” scale (Table 1). This latter evaluates social skills (4 items) and social roles (3 items).
    - Clinical Global Impression (CGI) - Severity and improvement scales
- ➢ At M3 (3 months ± 15 days)
  - ✓ Clinical and therapeutic aspects, including:
    - Treatment follow-up (number of missed injections, date and reason for discontinuation of treatment, if necessary)
    - Clinical Global Impression - Severity scale (CGI-S)
    - Adverse events (AE)
- ➢ At M6 (6 months ± 1 month) or termination of the study
  - ✓ Clinical and therapeutic aspects, including:
    - Treatment follow-up (number of missed injections, date and reason for discontinuation of treatment, if necessary)
    - PANSS core psychotic symptoms
    - Functioning, assessed by the GAF scale and by an ad hoc Social Functioning in Schizophrenia scale (Table 1).
    - Clinical Global Impression (CGI) - Severity and improvement scales
    - AE

### Outcome data analysis

#### Primary endpoint

Global functioning in patients remitted (*N* = 374) and non-remitted (*N* = 976) at M6. Remission was evaluated using the consensus RSWG criteria, i.e.,: a state of no greater than “mild” intensity in core psychotic symptoms, sustained for a minimum duration of six months [4]. As per DSM-IV (see ref. [18]), a score of 60 out of 100 was taken as a cut-off to identify patients with moderate to severe impairment in functioning (GAF score ≤ 60, representing some mild difficulty in socio-professional activities or satisfactory activity).

Missing data concerning PANSS and/or GAF data at M0 and/or M6 were considered as major deviations to the protocol.

#### Secondary endpoints

- ➢ Social functioning: evolution, comparison of social functioning between remitted and non-remitted patients at M6
- ➢ Social abilities as a function of psychosocial measures
- ➢ AE in “real-life”

### Adverse events

Adverse events (AE) were reported in the case report forms beginning at the inclusion of the study (M0) until one month after the study was finished. AE appearing with a delay that was chronologically consistent with the administration of the product were considered to be related to risperidone LAI treatment when a causal relationship was possible, likely, or very likely:

- ➢ *Possible relation:* The AE can also be attributed to a concomitant disease or other medications. Causality cannot be excluded.
- ➢ *Likely relation:* The attribution to a concomitant disease or the other medications does not seem likely, and risperidone LAI treatment discontinuation led to the disappearance of the AE or improved symptoms.
- ➢ *Very likely relation:* The AE cannot be attributed to a concomitant disease or other medication. The time to onset was chronologically very consistent with the administration of risperidone LAI, risperidone LAI treatment discontinuation led to the disappearance of the AE or improved symptoms, and risperidone LAI re-administration led to a recurrence of the same AE symptoms.

### Patient population size

We estimated that an initial sample size of 1500 patients was required to have ≥80 % power in detecting a difference in outcome of ≥10 % (if the two populations were relatively similar in size and that the smallest comprises 35 % or more of the total survey population).

### Statistics

Statistical comparisons between the two groups of patients were done by using the following tests: Mantel-Haenszel Chi-2 test for stratified categorical variables, Chi-2 of the homogeneity test for non-stratified categorical variables, and the non-parametric Wilcoxon test for continuous variables. The statistical level of significance was accepted for *p* values < 0.05. Values are presented as mean ± SEM (standard error of the mean).

## Results

The study included 1491 patients at M0 (security safety analysis set). One patient was excluded because of a major protocol deviation (no PANSS or GAF data available from M0 or M6). Of the remaining 1490 patients, 1408 (94.5 %) and 1354 (90.9 %) were seen again at M3 and M6, respectively. The main reason for discontinuation was patients’ decision (68.6 %). Follow-up time was 5.6 ± 1.0 (mean ± SD).

### Demographic aspects and medical history of the participating patients

Table 2 shows demography and psychiatric history of the patients at M0 and M6. At M0, patients were mostly men (65.2 %) and their average age was 36.7 ± 0.3 years. Most of them were single (69 % of patients) and without children (72 % of patients). More than half were on disability or sick leave for psychiatric disorders (56 % of patients). More than three-quarters of patients (76.4 %) were diagnosed as schizophrenia disorder according to the DSM-IV criteria (Table 2). The disease duration was ≤ 10 years for 62.5 % of the patients.

**Table 2:** Demographic aspects and medical history of the included patients

	Month 0	Month 6
Number of patients	1490	1354
Age (years)	36.7 ± 0.3	36.8 ± 10.9
Sex (% males)	65.2 %	64.6 %
Marital status (% patients)		
Married or cohabiting	17.2 %	16.9 %
Single	68.9 %	68.8 %
Divorced, widowed, separated	13.9 %	14.3 %
Patients with children (% patients)	27.6 %	27.8 %
Accommodation (% patients)		
Independent housing	62.6	63.1
Dependent housing	20.9	20.7
Homeless housing	1.3 %	1.3 %
Hotel	0.9 %	1.0 %
Institutional center/Foundation	8.9 %	8.7 %
Retiring home	0.5 %	0.6 %
Other	4.8 %	4.7 %
Current employment status (% patients)		
Employee	12.2 %	11.7 %
Student or in training	6.3 %	5.9 %
Help Center for Labor	7.2 %	7.3 %
Unemployed or retired	9.6 %	9.5 %
Freelancer	1.0 %	1.1 %
Disabled or sick leave for psychiatric disorder	56.4 %	57.0 %
Status unknown	0.8 %	0.9 %
Other	6.5 %	6.5 %
Psychiatric history		
DSM-IV diagnostic (% patients)		
Schizophrenia	76.4 %	76.4 %
Schizophreniform disorder	23.6 %	23.6 %
Chronicity (% patients)		
≤ 5 years	30.7 %	30.3 %
> 5 and ≤ 10 years	31.8 %	31.9 %
> 10 and ≤ 15 years	16.6 %	16.6 %
>15 years	20.8 %	21.2 %

Values are given as mean ± SEM or as % of patients. M0, month 0 (inclusion). M6, month 6 (end of study)

### Clinical assessment

**Fig. 2:**
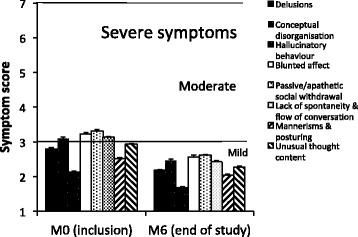
PANSS core psychotic symptom scores at M0 and M6

Figure 2 shows PANSS core psychotic symptom scores at M0 and M6. At M0, four mean rating score values were of moderate severity (conceptual disorganization, blunted affect, passive/apathetic social withdrawal, lack of spontaneity and flow of conversation). At M6, all mean rating score were of mild severity.

**Fig. 3:**
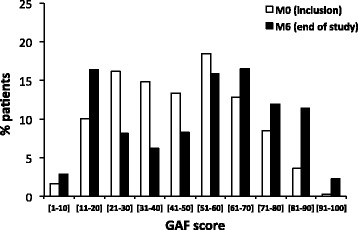
Frequency distributions for global functioning scores at M0 and M6

Table 3 shows that the mean global functioning (GAF rating score) significantly improved (*p* < 0.001) by 5.8 ± 0.45 from M0 (46.4) to M6 (52.2). Figure 3 shows frequency distributions for global functioning scores at M0 and M6. The percentage of high functioning patients (GAF rating scores >60) increased from 25.2 % at M0 to 42.1 % at M6 (*p* < 0.0001).

**Table 3:** Evolution of functional performance between M0 and M6

	M0	M6	M6-M0	*p*
	(*N* = 1490)	(*N* = 1354)		
GAF rating score^a^	46.4 ± 0.54	52.2 ± 0.67	5.8 ± 0.45	< 0.001
Social functioning rating score^b^	18.3 ± 0.11	20.2 ± 0.12	1.9 ± 0.10	< 0.001
Social skills	11.4 ± 0.06	12.5 ± 0.07	1.1 ± 0.07	< 0.001
Social roles	6.90 ± 0.05	7.73 ± 0.06	0.83 ± 0.05	< 0.001

Values are given as mean ± SEM. M0, month 0 (inclusion). M6, month 6 (end of study)

^a^Maximum GAF rating score = 100

^b^Social functioning rating scores = total (from 7 to 28), social skills (from 4 to 6) and social roles (from 3 to 12)

Social functioning significantly improved between M0 and M6 (Table 3). Thus, mean social functioning total scores significantly increased by 1.9 ± 1.0 from 18.3 to 20.2 (*p* < 0.001). This resulted from a significant increase (*p* < 0.001) in the rating scores of both social skills and social roles (Table 3).

CGI rating scores showed a tendency to decrease throughout the observation period. The average scale score “CGI - Severity” slightly decreased from 4.0 ± 0.032 at M0, to 3.6 ± 0.032 at M3 and 3.3 ± 0.035 at M6, and the average scale score “CGI - Improvement” evolved from 2.4 ± 0.022 at M0 to 2.2 ± 0.027 at M6. Consistently, the percentage of patients with CGI severity scores ≥ 5 (categorized “markedly ill”, “severely ill” or “among the most extremely ill patients”) decreased from 37.9 % at M0, to 21.3 % at M3 and to 18.7 % at M6.

### Other treatments

The majority of the patients (70.8 %) had another psychotropic medication, particularly anxiolytics (57.6 % of patients), hypnotics (41.8 %) and sedatives (27.0 %). This propensity of patients for other psychotropic medications remained high at M3 (64.6 % of patients) and M6 (62.8 %).

Concerning psychosocial measures, non-specific intervention support was implemented for more than half of the patients (55.7 %, 53.5 % and 55.9 % at M0, M3 and M6, respectively).

### Remission status according to the PANSS scale (core psychotic symptoms)

**Fig. 4:**
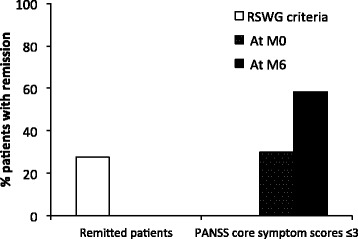
Remission status according to the PANSS scale (core psychotic symptoms). RSWG criteria include: a score ≤ 3 for the 8 items of the PANSS scale maintained for at least 6 months. This time criterion could not be evaluated at M0 due to the lack of data before inclusion. However, the PANSS score was first evaluated at M0 and then 6 months later at M6, so it was possible to evaluate the proportion of patients who met the RSWG criteria at M6

Figure 4 shows that 27.7 % of the patients were in remission at M6, according to the RSWG criteria. This compares well with the 29.9 % of patients with “mild” intensity in core psychotic symptoms at M0, but not with the 58.4 % of patients with “mild” intensity in core psychotic symptoms at M6 (Fig. 4).

At the inclusion visit, patients with a remission of schizophrenia had the following characteristics, as compared with non-remitted patients:

- ➢ Better global functioning (GAF rating score = 53.8 ± 1.4 vs. 43.6 ± 0.54, *p* < 0.001)
- ➢ A better social functioning profile (total score = 21.0 ± 0.19 vs. 17.2 ± 0.12, *p* < 0.001)
- ➢ A better CGI severity score (3.0 ± 0.052 vs. 4.4 ± 0.032)
- ➢ A better CGI improvement score (1.9 ± 0.036 vs. 2.6 ± 0.022)

### Functional performance in remitted schizophrenia patients

Table 4 shows the functional performance at M6 in patients with remission of schizophrenia. The global functioning rating score was 62.5 ± 1.5 (the main objective of the study). This was significantly higher than the mean GAF score in non-remitted patients (48.3 ± 0.74, *p* < 0.001). Social functioning rating scores were also significantly higher (*p* < 0.001) in remitted patients (Table 4).

**Table 4:** Functional performance at M6, in remitted schizophrenic patients (RSWG-criteria)

	Remitted patients	Non-remitted patients	*p*
Number of patients	374 (27.7 %)	976 (72.3 %)	
GAF total rating score^a^	62.5 ± 1.5	48.3 ± 0.74	< 0.001
Social functioning			
Total rating score^b^	21.0 ± 0.19	17.2 ± 0.12	<0.001
Social skills rating score^b^	12.8 ± 0.11	10.8 ± 0.07	<0.001
Social roles rating score^b^	8.2 ± 0.10	6.4 ± 0.06	<0.001

Values are given as mean ± SEM or as % of patients. M6, month 6 (end of study)

^a^Maximum GAF rating score = 100

^b^Social functioning rating scores = total (from 7 to 28), social skills (from 4 to 16) and social roles (from 3 to 12)

### Adverse events

Risperidone LAI was well tolerated. Only 88 of 1491 patients (6 %) reported at least one AE (anxiety, insomnia, depression, headache). The investigator considered that 69 AE (18 severe) were related to risperidone LAI treatment.

## Discussion

We analysed a large sample in real-life conditions, with a long follow-up and low attrition rate. After one year of risperidone LAI treatment, 27.7 % of the study’s patients were in RSWG-remission. This high remission rate was quantitatively similar to that observed in the StoRMi trial (33 %) [15] and higher than that observed with oral risperidone in the CATIE study (6.3 %) [9]. This result can be likely explained by non-adherence to long-term oral medication regimes, which is one of the most significant therapeutic issues in the therapy of schizophrenia and related disorders [3].

Previous studies showed functional improvement under risperidone LAI treatment in patients with *stable* schizophrenia or schizoaffective disorder [11–14]. On the other hand, the mean GAF rating score in remitted patients (62.5) was higher than the cut-off previously used to identify patients with satisfactory functioning (60) and significantly higher than the mean GAF score in stable, non-remitted patients (48.3). Our study thus clearly shows that further improvement can be obtained in remitted patients (as compared with stable, non-remitted patients).

Achieving functional recovery is a key goal for clinicians treating patients with schizophrenia, but patients with schizophrenia and their families are also concerned with the regain of a patient’s ability to act or to give meaning to his or her life (empowerment) and social functioning (managing daily life, social and/or professional skills). It seems, therefore, appropriate to include social functioning as a supplementary criterion of recovery.

Our study suggested that social functioning is also higher in remitted patients as compared with non-remitted patients. However, our scale should be validated in further studies and/or other social functioning scales should be used to confirm this point.

It is important to mention that patients under risperidone LAI continued to improve between their 6 and 12 months of treatment. Thus, the percentage of patients with mild intensity in core psychotic symptoms (PANSS) increased from 29.9 to 58.4 % between 6 and 12 months treatment. In the same period, the percentage of high functioning patients (GAF rating scores >60) increased from 25.2 to 42.1 %. The percentage of patients with CGI severity scores ≥ 5 (categorized “markedly ill”, “severely ill” or “among the most extremely ill patients”) decreased from 37.9 % at M0, to 21.3 % at M3 and to 18.7 % at M6. These results illustrate the importance of long-acting antipsychotic treatment for ensuring a continuity of symptomatic and functional remission.

Risperidone LAI should be investigated for other recovery aspects of schizophrenia, since patients in RSWG-remission have a better cognitive outcome [19, 20] and quality of life [21, 22], but may perceive a decreased sense of wellbeing [23]. In addition, Rocca et al. [24] reported that second generation antipsychotic use predicts better social functioning and better executive functions.

### Limitations of the study

The results apply to chronic patients, two-thirds of them with over 5 years of the disorder. The scope of our study does not allow for a certain conclusion about symptom improvement being due to risperidone specifically, since there is no information about other treatments. Furthermore, the choice of a GAF of 60 as cut-off for functional remission was frequently used, but it is arbitrary. Finally, the Social Functioning in Schizophrenia is an ad hoc scale. Such limitations should be taken into consideration by future studies.

## Conclusions

Our results clearly show that after one year of treatment with risperidone LAI, RSWG-remitted patients have a high level of global functioning, which is significantly higher than in stable, non-remitted patients. Social functioning was also higher in remitted patients as compared with stable, non-remitted patients. This latter should be confirmed in further studies using validated social functioning scales.

## Availability of data and materials

Data and materials can be requested to Dr. Ricardo P. Garay (ricardo.garay@orange.fr).

## Abbreviations

AE: Adverse Events
CCTIRS: Comité Consultatif pour le Traitement de l’Information en matière de Recherche dans le domaine de la Santé (Advisory Committee for the Evaluation of Information in the field of Health Research)
CEGEDIM: Centre de gestion et de Documentation de l’Information Médicale (Management and documentation centre of medical information)
CGI: Clinical Global Impression
CGI-S: Clinical Global Impression - Severity scale
CNIL: Commission Nationale de l’Informatique et des Libertés (National Committee of informatics and Freedom)
EVeREST: EValuation of functioning in REmission after Symptomatic Treatment
GAF: Global Assessment of Functioning
LAI: Long-Acting Injection
PANSS: Positive And Negative Symptom Scale
RSWG: Remission in Schizophrenia Working Group
StoRMi: Switch to Risperidone Microspheres
SEM: Standard Error of the Mean
